# Hormesis as a Hidden Hand in Global Environmental Change?

**DOI:** 10.1021/acs.est.5c01101

**Published:** 2025-02-05

**Authors:** Matthias C. Rillig, Anika Lehmann, Mohan Bi

**Affiliations:** † Freie Universität Berlin, Institute of Biology, 14195 Berlin, Germany; ‡ Berlin-Brandenburg Institute of Advanced Biodiversity Research, 14195 Berlin, Germany

**Keywords:** hormesis, low-dose effects, global environmental change, climate change, chemical pollution

Humans have produced and are releasing a staggering amount of chemicals into the environment, with one study estimating that there are at least 350 000 chemicals and mixtures being produced industrially.^1^ Many of these substances are environmentally available, if only at very low concentrations, and may be toxic, with potential toxicity known for only a tiny fraction of this list of potential pollutants. This low-dose occurrence of a potentially large number of substances, at various temporally dynamic compositions in probably all ecosystems, gives rise to an intriguing and largely unstudied possibility: that these substances give rise to hormetic responses in ecosystems. Hormesis describes a positive response of an organism to very low doses of a toxicant while higher doses induce negative effects (Figure 1A).^2,3^

**Figure 1:**
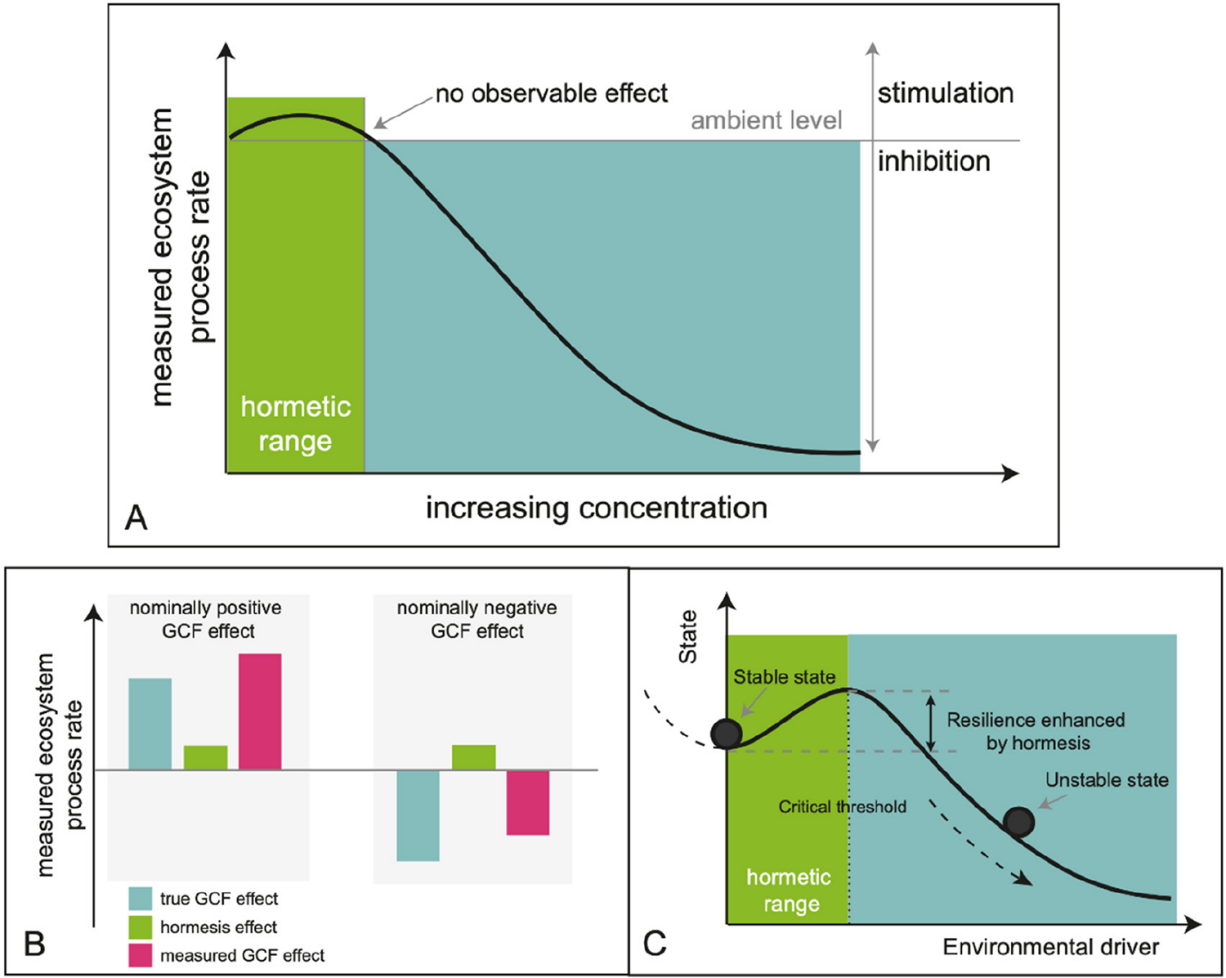
(A) Hormetic responses mean a response to a negative influence is positive at very low doses. (B) If widespread, such hermetic effects could lead to ecosystem process rates being affected, leading to an overestimation or underestimation of the effect of other factors of global change. (C) When environmental drivers are exceeding the hormetic threshold, the ecosystem state can undergo a transition from a stable state to an unstable state.

Hormesis is becoming increasingly well documented in a range of organisms, including plants, animals, and microbes,^4^ and is mechanistically understood in many cases, but still controversial in the field of human health.^5^ Recently, hormesis has also been observed at community and ecosystem levels, including forest ecosystems experiencing low levels of climate stressors,^6^ but how ecological communities respond to several co-occurring low-dose substances is generally not well-known. Are such low-dose putatively positive effects additive, synergistic, or antagonistic when a large number of substances are present?^7^ Importantly, different substances will cause hormetic responses in different organisms and under different settings, such that a higher diversity of low-level contaminants might trigger a greater number of low-dose positive responses in a larger proportion of an ecosystem’s biodiversity. It is therefore possible that widespread, low-level environmental contamination leads to various short-term stimulatory responses, enhancing ecosystem functions.

## Hormesis and Global Environmental Change

Human-caused environmental effects are often not subtle, collectively known as global environmental change,^8^ consisting of factors such as climate change, increased atmospheric levels of carbon dioxide, plastic pollution, invasive species, heavy metals, and organic chemicals. Deleterious effects of such global change factors on organisms, communities, and ecosystem processes are well-documented^8,9^ and typically address larger effect sizes arising from higher concentrations, in the case of chemical factors. Could the ever-present background of potentially positive responses caused by widespread low-level chemical contamination ameliorate responses to other factors of global change? This would run counter to the current expectation that chemical pollution only worsens effects of other anthropogenic influences,^10^ based on the notion that chemical pollutants represent additional pressures to organisms and ecosystems, an idea that has increasing empirical support, albeit based on higher concentrations of pollutants. Testing this hypothesis about hormesis is important, because we may have underestimated the true extent of other global change effects (Figure 1B), meaning the effects of many other factors of global change (including climate change) with their often negative consequences on biota and ecosystems could be partially masked by these stimulatory background effects. Analogously, global change factors with nominally positive effects on certain process components (such as temperature or nitrogen deposition) could have been overestimated in their effects if the stimulation stemming from hormesis were a part of this response.

The hormetic effects of various environmental factors may contribute to the emergence of tipping points in ecosystems in response to global change (Figure 1C). Similar to how low-dose chemical exposures stimulate cellular protective mechanisms, hormesis in ecological contexts can enhance organismal performance and resilience to environmental stressors,^6^ potentially mitigating the collective impact of multiple stress factors. However, as the number or intensity of these low-dose stressors exceeds the hormetic threshold, the overall system response may shift dramatically. This transition occurs due to not only the disappearance of the protective “masking effect” but also the cumulative negative impacts of previously beneficial stressors. Therefore, understanding the hormetic effects of environmental factors is crucial for identifying early warning signals of potential ecosystem tipping points.

It is important to emphasize that both observational and experimental work in the environmental sciences would be affected by such unknown hormesis effects. The case is clear for observational studies, because the low-concentration effects would typically go unnoticed in the study of dominant factors, and in many surveys, such low-level concentrations of a wide range of background chemical pollutants would not even be measured. The same, however, is also true for highly mechanistic experimental work, because in the Anthropocene there will inevitably be a background of contamination in any environmental substrate (the ambient level),^11^ except for in the most artificial circumstances.

## Open Questions

While this is perhaps a surprising twist to the story of environmental pollution, there are very many open questions. The most important one is clearly if this amelioration of global change effects by hormesis really does exist, and if so what its limits are, especially given the large number of toxicants and an equally growing list of other factors of global change. At what critical point, with regard to concentration, toxicity, and exposure duration to such a pollutant background, does the potentially ameliorating effect of the low-concentration pollution flip to become an additional burden for organisms? Can short-term stimulatory effects really help with long-term stressors that are characteristic of global change, including factors of climate change? To what extent do these stimulatory effects on organisms propagate to the level of biological communities and their composition and to measurable ecosystem process rates? If effects do propagate to the ecosystem level, it seems unlikely that all functions of aquatic or terrestrial ecosystems are affected equally, and thus, it is important to study which ecosystem processes are particularly affected and why. It seems entirely possible that some ecosystem processes (or compartments) will be experiencing a stimulation, whereas for others, the presence of these background pollutants has negative consequences.

## Conclusions and the Way Forward

To address all of these questions, a concerted international research effort will likely be needed. The path forward should be a combination of experiments, observational studies, and modeling. Carefully executed experiments will be needed to establish causality of effects and to study underlying mechanisms of potential hormesis. Importantly, these should be conducted at various levels of complexity, from in vitro lab experiments to mesocosms allowing access to ecosystem dynamics. Such experiments should be complemented by large-scale observational studies based on high-resolution environmental analytics, which, using machine learning, could be used to test for the existence of signals of hormesis across broad environmental and biogeographical gradients. Modeling can be used to integrate findings of experiments and observational data and to inform risk assessments, and to formulate predictions of future impacts. In this work, it will also be important to carefully control the narrative, especially in communicating with the public. Under no circumstances should these hormesis effects be regarded as a “silver lining”, something positive, in the sense of being desirable. These are still all pollutant effects, just that in a particular concentration range and situation they may be stimulatory; thus, it will always be important to reduce the levels of these pollutants as much as possible. A necessary first step will be an open mind about studying hormesis, and for researchers to accept the operational risk of using very low doses of chemicals. Widespread adoption of this research by environmental scientists may unlock critical insights into planetary health.
